# Modal Damping Coefficient Estimation of Carbon-Fiber-Reinforced Plastic Material Considering Temperature Condition

**DOI:** 10.3390/ma13122872

**Published:** 2020-06-26

**Authors:** Ho-Young Kang, Chan-Jung Kim, Jaewoong Lee

**Affiliations:** 1 Rail&Vehicle Technology Team, Smart Manufacturing Innovation Center, #8, Jisiksaneop5-ro, Hayang-eup, Gyeongbuk 38408, Korea; hykang@gbtp.or.kr; 2 Department of Mechanical Design Engineering, Pukyong National University, 45 Yongso-ro, Nam-gu 48513, Korea; 3 Department of Fiber System Engineering, Yeungnam University, Gyeongsansi, Gyeongsanbokdo 38541, Korea

**Keywords:** modal damping coefficient, temperature condition, direction of carbon fiber, resonance frequency, carbon-fiber-reinforced plastic material

## Abstract

Excellent mechanical properties of carbon-fiber-reinforced plastic material (CFRP) demonstrates many possibilities in industries using lightweight materials, but unlike isotropic materials, such as iron, aluminum, and magnesium, they show direction-sensitive properties, which makes it difficult to apply for them. The sensitivity of a modal damping coefficient of a CFRP material over the direction of carbon fiber was examined on spectral input patterns in recent research, but the effect of temperature was not considered up to now. To overcome this, uniaxial vibration tests were conducted using five simple specimens with different direction of carbon fiber in a CFRP specimen, the frequency response functions were experimentally determined and the modal damping coefficients were calculated. It was revealed that the resonance point and the modal damping of the specimen changed according to the change in temperature condition. Based on the experimental results, it was demonstrated that the theoretical frequency response function of the carbon composite material is a function of temperature, and it was confirmed that the nonlinear characteristic of the modal damping was the smallest under the 0 degree of direction of carbon fiber.

## 1. Introduction

A damping action cancels out external load energy by converting it to internal energy using the inherent properties of the material, and is beneficial in terms of durability as it increases structural safety and reduces response. A damping coefficient is used in a time domain or frequency domain to express a damping action, and accurate identification of its value plays a very important role in understanding mechanical properties of a target system, and various methodologies have been proposed. In particular, in order to express damping in a frequency domain, a frequency damping value corresponding to each resonance point is required, which is referred to as a modal damping coefficient [1,2]. In order to obtain the frequency damping value, the value is usually identified through a modal test using an impact hammer, etc., but the physical quantity is vulnerable to external noise, and thus the average value through repeated tests is usually used. The modal test method for measuring the modal damping coefficient has a weakness that it is vulnerable to noise and thus only an approximate value can be found, as mentioned above. In addition, since the method is performed under limited conditions in which the input excitation type is dominated by a specific excitation pattern such as impact, the method is inevitably subject to a large error under various actual input patterns if the modal damping value is affected by the external input pattern. Accordingly, developing a method for identifying modal damping coefficient values considering the effects of input patterns of external loads has a very important meaning in securing the mechanical reliability of materials.

Carbon-fiber-reinforced plastic (CFRP), which has excellent specific strength characteristics, is a next-generation lightweight material that can replace existing steel materials and aluminum materials, and basic research and product development on it are actively being conducted [3,4,5,6,7,8,9]. Since the product industry that utilizes the product is limited to only some transportation modes such as bicycles and to a small amount thereof, specialized producers are performing production in a custom-made fashion by hand. Accordingly, many technical difficulties, such as low product uniformity for prototypes and the need to develop efficient processes for mass production, have yet to be overcome in order for the product to be applied to mass production industries such as the automobile industry. Mechanical properties of the carbon composite material basically depend on the conditions of the carbon fiber and the polymer resin that make up the product. In addition, what fabrics are combined to form the carbon fiber greatly affects the mechanical properties of the product. In general, there are various candidates for the main fabrics used for carbon composite materials, such as plain weave, twill weave, and unidirectional, and thus there are various laminated structures in which these are combined. Therefore, in order to utilize the carbon composite material in products, it should be noted that the mechanical properties basically vary depending on the laminated structure, and the test results necessarily vary as well [10,11,12].

Modal damping is a very important mechanical property in terms of system stability, as mentioned above, and the conditions equally apply to carbon composite materials. In particular, damping characteristics of carbon composite materials show relatively large values compared to those of other steel materials, and thus they are attracting great attention as materials for parts to be applied for mechanical products, in addition to their superiority in terms of specific strength. Accordingly, when carbon composite materials exhibit different damping characteristics depending on the excitation profile conditions, they inevitably have a large error compared to other materials due to the difference in absolute values. Therefore, in this experiment, we will describe how we derived the damping coefficient by using two half-power points that could be observed near each resonance point of the frequency response function used in existing steel products, etc. In particular, we performed tests while setting boundary conditions of the carbon composite material for measuring the modal coefficients to be the simplest form in which one side was completely fixed, so that the reproducibility of the test results could be high. In a recent study, analysis of the sensitivity of the carbon composite material specimens to different frequency pattern inputs was conducted using the frequency response function, but changes in the modal damping characteristics with respect to the temperature variable were not investigated [12]. The sensitivity analysis of mechanical system can derive design guideline for engineers under the minimum design modification policy [13,14].

In this study, changes in the mechanical properties of the carbon composite material, which is a representative lightweight material, under various temperature conditions were investigated through uniaxial excitation tests. The theoretical frequency response function of CFRP simple material was proposed by assigning two parameters, resonance frequency and modal damping coefficient, as function of temperature condition. Both resonance frequency and modal damping coefficient were obtained through the calculated frequency response function at uniaxial excitation test for five CFRP specimens under different direction of carbon fiber, 0, 30, 45, 60, and 90 degrees. Based on the results of the modal damping measurement test for specimens with different directions, we evaluated the sensitivity of CFRP specimens over a temperature condition on the modal damping results. In addition, the characteristics of modal damping for different direction of carbon fiber were discussed from the experimental consequences. However, the physical reason why the dynamic parameters, resonance frequency, and modal damping coefficient were a function of the temperature condition and have a different variation trend for a different direction of carbon fiber was not considered in this study because this study was focused on the finding the sensitivity of the CFRP material over the temperature condition only.

## 2. Estimation Method for Modal Damping Coefficient

A typical method of measuring a modal damping value is basically performed through a modal test using an impact hammer to obtain the value. After exciting the measurement target with an impact hammer and obtaining the frequency response function using the acceleration data measured at the position where the response occurs, the damping coefficient at the main frequency is derived using the slope of the cusp near the resonance point of the measured frequency response function. This method is performed under the assumption that the target product shows linearity with respect to the external load because the method uses the frequency response function. In the case of a product with a large nonlinear characteristic, in order to perform a more rigorous experiment, the response characteristics at different frequencies are measured in detail through harmonic excitation in which measurement is performed while raising or lowering the frequency using an exciter, instead of the above-mentioned impact excitation, and then the modal damping coefficient at the resonance point is obtained. The foremost advantage of this method is that it is a method of applying an impact using an impact hammer, and thus it does not affect any dynamic characteristics of the target object because it does not involve any contact with the target object (contact herein refers to a constant connection). However, since the impact excitation method uses an input signal having all frequency components, which are simultaneously inputs, the method has critical disadvantages that distortion may occur in a product having a large nonlinear characteristic, and that the excitation pattern cannot be changed.

In this study, a method of introducing an exciter-based excitation method with limitations of boundary conditions in order to control the excitation source as precisely as possible and to obtain a frequency response function through numerous iterative tests was used. An advantage of the exciter is that desired excitation profiles can be guaranteed under excitation conditions with reliability and little error. In particular, in the excitation conditions of this test method, in addition to the method of simultaneously exciting all frequency bands using an existing impact hammer, the method of sequentially increasing the excitation frequency can be utilized as well. In the former case, the excitation profile is a random excitation in which all frequency values are simultaneously applied within the desired frequency range. In this excitation method, it is possible that some distortion may occur for a target object having nonlinear characteristics. On the other hand, if the starting frequency, the ending frequency, and the slope at which the frequency gradually rises are selected after the entire frequency band is determined, a single frequency can be applied sequentially by automatically raising or lowering the excitation frequency. This test method has a disadvantage that the test is in the form of applying one frequency and receiving a response thereto and thus is performed over a long time. Other than that, it is a method by which the response at the frequency can be received with the highest reliability. Therefore, in this test, a process of measuring the damping characteristic value for the target object, the carbon composite material, was performed using a method of applying harmonic excitation by utilizing a uniaxial exciter.

**Figure 1:**
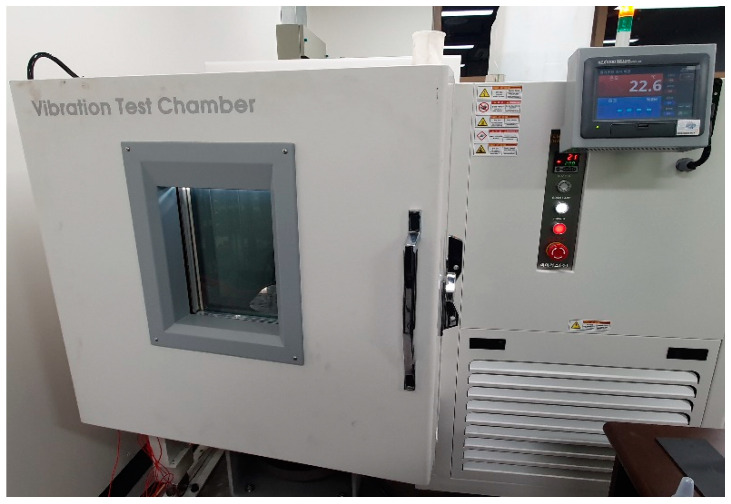
Configuration of the temperature chamber of the uniaxial exciter.

In order to control the temperature condition, a chamber was used to maintain the intended temperature during the excitation test of CFRP materials. The controller of exciter was operated separately from the controller of temperature chamber and the excitation test was conducted 10 min later after reaching target temperature because the controlled room temperature may be different from the temperature at the responsible CFRP specimen. The configuration of the temperature chamber is illustrated in Figure 1.

The damping coefficient of a structure was obtained by using a method, which is performed under the assumption that the damping coefficient is a fixed value, and which utilizes the frequency response function. The modal damping value is a very important factor in physically determining the magnitude of the response in the resonance point section and, at the same time, is commonly used in theoretical equations and analysis models through identification of the value. First, the ratio of the response (*R*(ω)) to the external excitation input (*F*(ω)) in the theoretical equation part is expressed as Equation (1) under the condition of an input frequency of ω (Hz).

(1) $$\frac{R\left(\omega \right)}{F\left(\omega \right)}=\overset{N}{\underset{i=1}{\sum }}\frac{R_{i}^{e}}{-M_{i}\omega^{2}+C_{i}\omega j+K_{i}}\;$$

Here, the $R_{i}^{e}$, $M_{i}$, $C_{i},$ and $K_{i}$ values represent the numerator component of the frequency response function, mass, damping, and stiffness in the i-th mode, respectively, and usually have one constant value. In addition, N represents the total number of modes and *j* denotes imaginary unit. The $C_{i}$ value can be measured or obtained using material property information. The modal damping coefficient is generally expressed as a modal damping (%) value suitable for representation in the frequency domain, rather than actually using the physical values. First, when Equation (1) is converted to a modal coordinate system, it can be expressed as Equation (2) below, and the transfer function is represented by $H_{i}\left(\omega \right)$ for the *i*-th mode.

(2) $$H_{i}\left(\omega \right)=\frac{r_{i}^{e}}{{\left(\omega_{n,i}\right)}^{2}-\omega^{2}+2\omega \omega_{n,i}\zeta j}$$

Here, $\omega_{n,i}$, $\zeta_{i},$ and $r_{i}^{e}$ values represent the resonance frequency, modal damping ratio, and normalized residual in the i-th mode, respectively. The corresponding function value is the most common value corresponding to a single input and single output model of a typical linear system. However, the characteristics of the product to be tested in this study depend on the temperature condition and the angle of the carbon composite material. If the variables expressing the conditions are T and θ, respectively, the frequency response function of Equation (1) is changed as follows.

(3) $$H\left(\omega ,\theta ,T\right)=\overset{N}{\underset{i=1}{\sum }}\frac{r_{i}^{e}}{{\left(\omega_{n,i}\left(\theta ,T\right)\right)}^{2}-\omega^{2}+2\omega \omega_{n,i}\left(\theta ,T\right)\zeta \left(\theta ,T\right)j}$$

The frequency response function condition in Equation (3) is a value that is limited to carbon composite materials, and does not correspond to general material properties. Therefore, the matter to be identified in this study is to perform an analysis after measuring the sensitivity to three variables under the frequency response function condition of Equation (3) through uniaxial excitation tests. Through this, the exact value of Equation (3) can be obtained under different conditions, and it can be seen that the modal damping characteristic is a function value that can be changed by the three variables. Another consideration with respect to the frequency response function is to convert the frequency response function values obtained at different locations into representative frequency response function values. If the frequency response function value measured at the response position k is $H^{k}\left(\omega ,\;\theta ,\;T\right)$ using the condition of Equation (3), the representative frequency response function $\hat{H}\left(\omega ,\theta ,T\right)$ for calculating the modal damping value is represented by the following Equation (4). Each frequency value is linearly summed and represented as one representative value. This is a possible representative value under the assumption that linearity is guaranteed for the observed CFRP specimen.

(4) $$\hat{H}\left(\omega ,\theta ,T\right)=\overset{M}{\underset{k=1}{\sum }}H^{k}\left(\omega ,\theta ,T\right)\;\;$$

We used the measured frequency response function to obtain the representative frequency response function as shown in Equation (4), then we utilized the relationship between the resonance points and the half-power points as a method to obtain modal damping corresponding to each mode in the frequency domain. If the frequency response function is given, the damping value corresponding to the *i*-th resonance point ($\omega_{n,i}$,) is as shown in Equation (4). Here, $\omega_{n,i}^{\left(1\right)}$ and $\omega_{n,i}^{\left(2\right)}$ correspond to two half-power points whose energy value is halved with respect to the center frequency corresponding to the resonance point.

(5) $$\zeta_{i}=\frac{\left|\omega_{n,i}^{\left(2\right)}-\omega_{n,i}^{\left(1\right)}\right|}{2\omega_{n,i}}$$

By using the modal damping measurement method of Equation (4), the damping value for each mode of the carbon composite material can be measured, and the modal damping value varies depending on the resonance point. Assuming that the resonance point of the carbon composite material is affected by the directionality and that the value varies depending on the temperature condition, the modal damping value is inevitably dependent on temperature according to Equation (4), and Equation (3), which is the frequency response function related to the carbon composite material, is valid.

## 3. Uniaxial Excitation Test of CFRP Specimen

### 3.1. Test Set-Up

**Figure 2:**
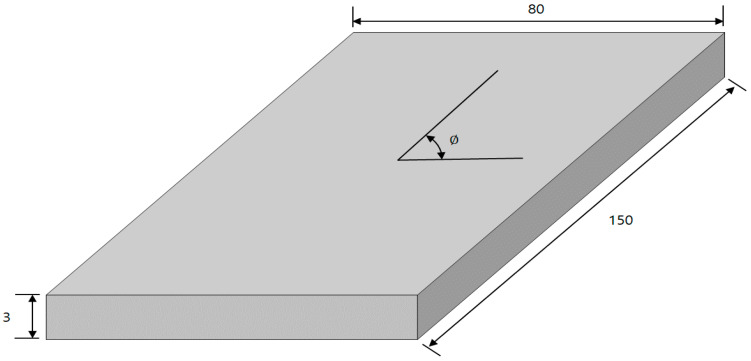
Configuration of five carbon-fiber-reinforced plastic material (CFRP) specimen (unit: mm) under different direction of carbon fiber: ϕ=0, 30, 45, 60, and 90 degrees.

Since the carbon composite material had directionality, damping coefficients were measured by performing vibration tests on specimens with various directionalities. For the carbon fiber, T700 from Toray industries, Inc. was used, and 12-layer laminated specimens of 3 mm height from SK Chemicals were prepared. Therefore, specimens with five directionalities were prepared and the physical properties of the carbon composite materials were the same. The five directionalities corresponded to 0 degrees, 30 degrees, 45 degrees, 60 degrees, and 90 degrees. Carbon composite materials basically have the same physical properties, but they will have very different stiffness in terms of directionality, so they inevitably have different dynamic properties when they are subject to vertical excitation. The shape of the specimen is a simple rectangular shape (80 mm × 150 mm) with a depth of 3 mm, and the angle is defined in Figure 2.

In order to find the damping characteristics of the carbon composite material, it is necessary to obtain a frequency response function corresponding to the ratio of the output to the input load. In order to obtain the corresponding conditions by using the exciter, one long side of the carbon specimen is tightly secured with a jig, and an exciter is attached to the fixed part to perform up-and-down excitation. Since the opposite side has a free boundary state, it can freely vibrate in one direction, and by measuring the response according to this relationship, a frequency response function due to vibration can be obtained.

The test environment for obtaining the frequency response function is the same, but as a method of applying a load through an exciter, the harmonic method of performing excitation while increasing one input frequency within a given time was used. For the temperature condition, a value between −8 and +105 °C was selected in consideration of the temperature control conditions of the chamber. Excitation frequency patterns and temperature conditions are summarized in Table 1 and Table 2, respectively.

**Figure 3:**
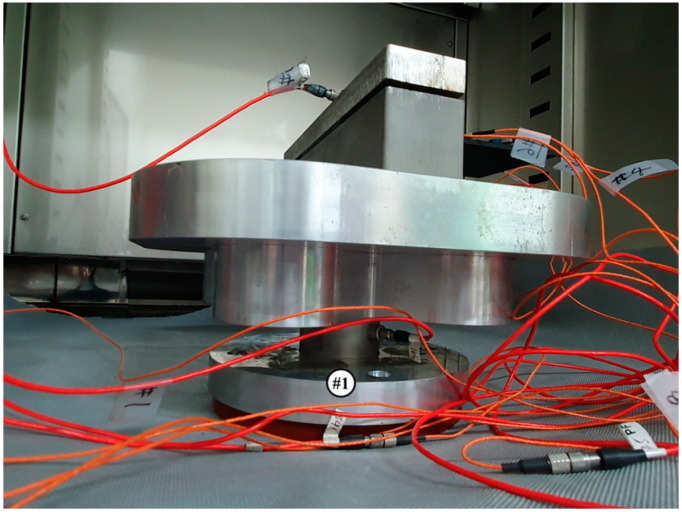
Location of force sensor on the CFRP specimen (ch#1).

**Figure 4:**
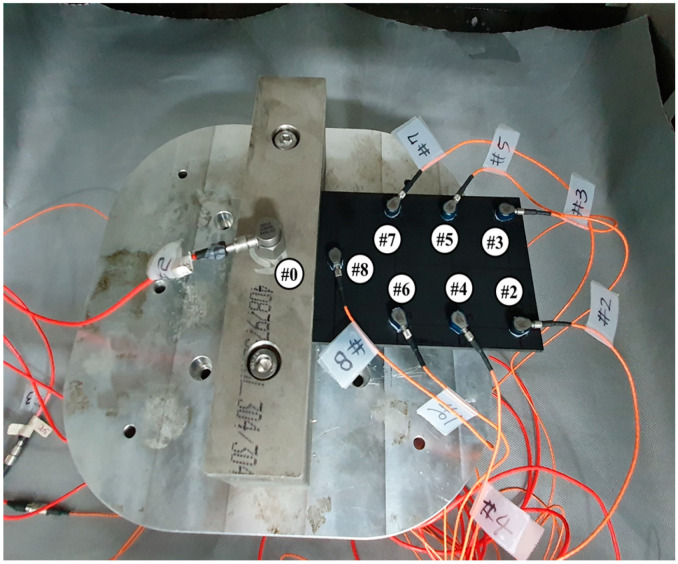
Location of accelerometers on the CFRP specimen (ch#2–ch8).

Under the above conditions, the vibration excitation test was performed, and a load sensor was attached to the bottom of the jig, which was excited at the same time, to collect the input excitation data, and an acceleration sensor was attached to the carbon composite material itself to collect the response acceleration at different locations. The frequency response function value can be obtained by converting both the load data for the input load and the data from the response acceleration sensor into frequency and then converting it in the frequency domain. Below are the measurement sensors. The reason for measuring the acceleration data at various locations is that the acceleration values at specific locations show a difference, so the acceleration sensors were acquired at various locations. Test.Lab from Siemens was used as the measurement equipment, and Dytran 1061V1 (see Figure 3) and 3225F20 (see Figure 4) were used as the load sensor and acceleration sensors, respectively. Since the excitation condition is up to 500 (Hz), the measurement was performed with the measurement sampling frequency condition of 1024 (Hz). In addition, the frequency response function was measured using the load data of channel 1 and the other 7 acceleration data, and under the conditions of Equation (3). Further, channel 0 was used as feedback data to control the excitation condition of the uniaxial exciter to implement the excitation condition selected above. Therefore, part of the jig that most accurately satisfies the excitation condition is the middle portion of the jig. The jig was fabricated by producing two rectangular jigs on one side of the carbon composite material, and then fixing the upper and lower jigs with two bolts. This jig should be designed/fabricated as a very rigid structure with rigid motion within the vibration frequency, and the fixed carbon composite material should be fixed very tightly, and therefore attention was paid to bolting. The material SUS304, which is believed to not cause plastic deformation even if the carbon composite material is clamped with a great force, was used as the material of the jig.

### 3.2. Test Results

**Figure 5:**
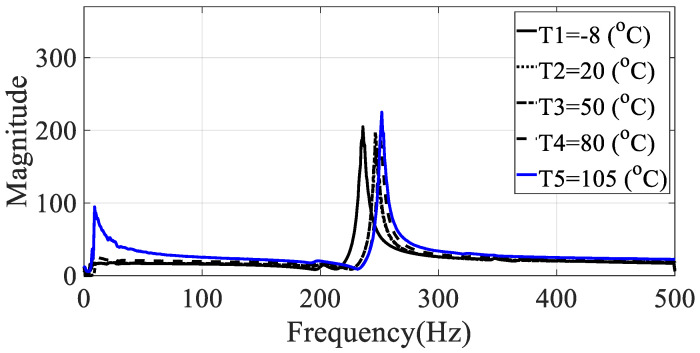
Representative frequency response function of specimen I (ϕ=0 degrees).

**Figure 6:**
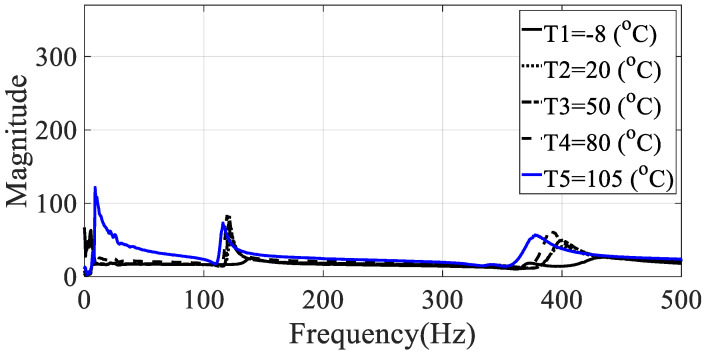
Representative frequency response function of specimen II (ϕ=30 degrees).

**Figure 7:**
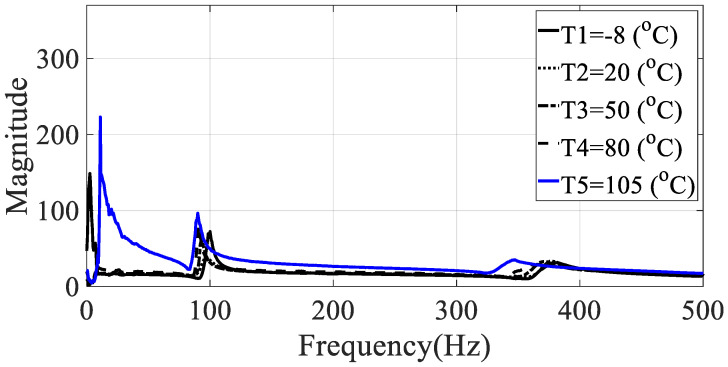
Representative frequency response function of specimen III (ϕ=45 degrees).

**Figure 8:**
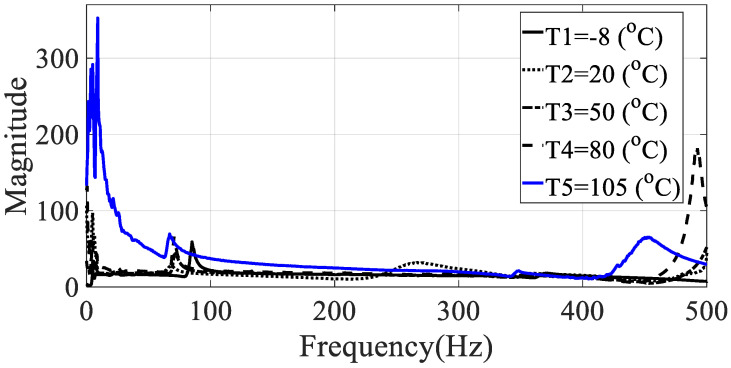
Representative frequency response function of specimen IV (ϕ=60 degrees).

**Figure 9:**
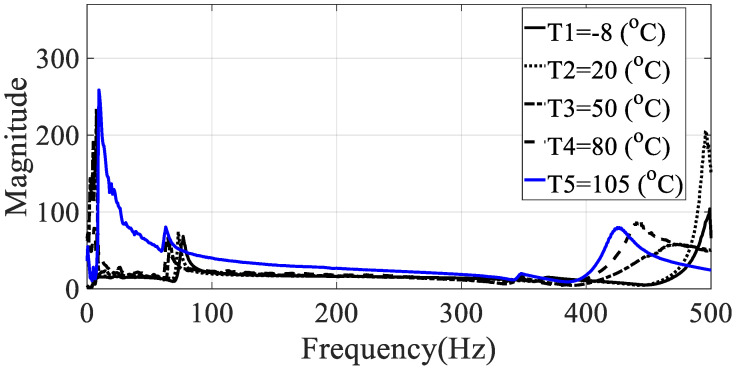
Representative frequency response function of specimen V (ϕ=90 degrees).

The frequency response functions for the five specimens (directionalities: 0, 30, 45, 60, and 90) were calculated using the relationship between the measured acceleration data and the load data. These values had the same excitation frequency range and show different characteristics depending on the excitation pattern. Since the vibration test was conducted with the harmonic excitation, single frequency components were input instantaneously and the input frequency components changed over time. Thus, the frequency response function was obtained in all frequency bands of interest by detecting the input frequency as a peak hold every 0.1 s. Seven frequency response functions were obtained from seven acceleration data, and the representative frequency response function of Equation (4) was obtained by using seven magnitude values corresponding to each frequency so that the modal damping coefficient could be obtained from one frequency response function, and the results for different specimens are shown in Figure 5, Figure 6, Figure 7, Figure 8 and Figure 9.

**Figure 10:**
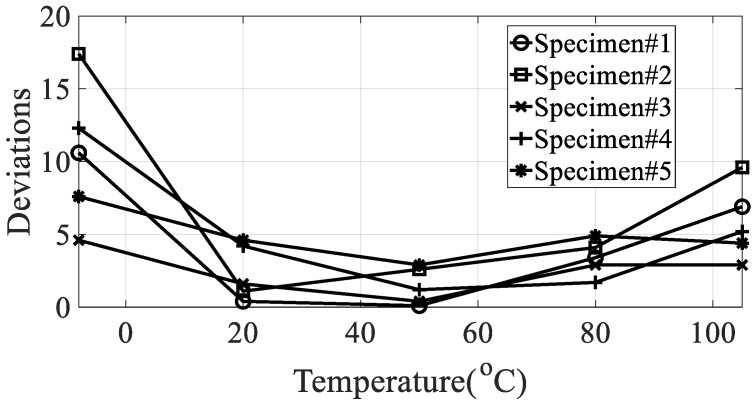
Deviation of the resonance frequency from the mean value according to different temperature conditions.

**Figure 11:**
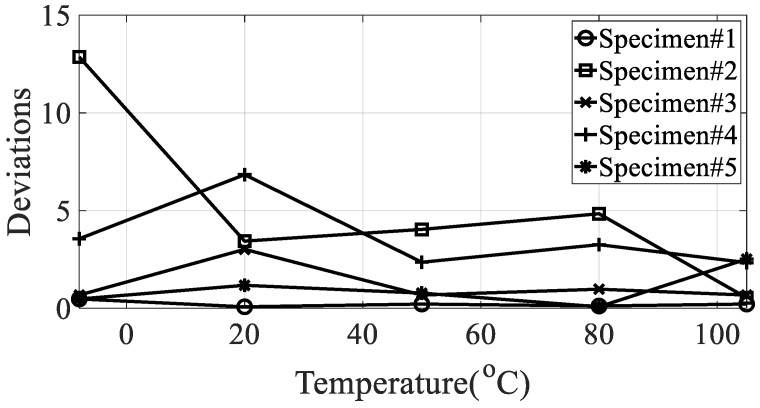
Deviation of the modal damping coefficient from the mean value according to different temperature conditions.

From the measured representative frequency response function, one rigid mode and two flexible models of CFRP specimens can be identified and the rigid mode about 10 Hz or less was not considered in this study because the sensitivity of dynamics variables in the frequency response function in Equation (3) was considered for flexible modes only. The rigid body mode may be dependent on static characteristics of mass or inertia of the CFRP specimen [1,2]. In order to calculate the modal damping coefficient, only components corresponding to the first mode of the representative frequency response functions were considered. The reason for considering this mode was that it was the main mode in which the mode shape appeared in the vertical direction, and it could be observed in all specimens within the frequency of interest. In the case of the second mode, comparison of the modal damping values under the conditions was not physically direct because the mode shape and the like appeared very different depending on the direction of the carbon composite material. Table 3 and Table 4 summarize the changes in the resonance point and the modal damping value of the first mode according to temperature change. In addition, the standard deviation (S.D.) in both parameters, the resonance frequency and the modal damping coefficient, was calculated to see the sensitive characteristics in different specimens. The variations in both parameters were illustrated in Figure 10 and Figure 11, respectively. It can be seen that both the resonance point and the modal damping value are affected by the direction and temperature of the specimen carbon fiber, and it can be seen experimentally that the theoretical frequency response function condition of Equation (3) is valid.

**Figure 12:**
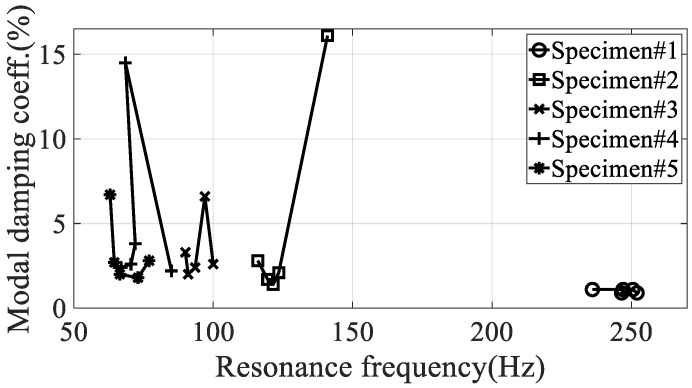
Relationship between resonance frequencies versus modal damping coefficients.

It can be seen that the resonance point increased or decreased as the temperature changed. It can be seen that, in the case of specimen #1, the resonance point increased, and in the case of other specimens, the resonance point decreased. The variation of resonance frequencies, the increase in specimen #1 or the decrease in other specimens, can be understood after investigating the mode shape of each mode case because the resonance frequency is closely related to the response behavior of responsible CFRP specimen. However, the research focus was investigate the sensitivity of the resonance frequency over different temperature condition so that the deviations from the mean value was only considered to evaluate the quantity of sensitivity rather than the minus or plus sign as shown in Figure 10. The modal damping value, unlike the resonance point, showed no consistently increasing or decreasing trend with increasing temperature, and very non-linear results were obtained. The only consistent fact is the deviation of modal damping coefficient is minimum at specimen #1 in every temperature condition as compared to other specimens. Table 5 shows the maximum relative error with the initial value of a condition of −15 °C for the same specimen. Additionally, the relationship conditions for judging the relationship between the resonance point and the modal damping coefficient are shown in Figure 12.

Considering the test conditions to fix one side and apply up-and-down excitation, specimen #1 was most likely to be selected because the first resonance point was higher than those of other specimens. The relative errors in both the resonance frequency and the modal damping coefficient also showed the specimen #1 was the smallest value over the variation of temperature condition so that the dynamic characteristic of the specimen #1 was superior to other specimens at the uniaxial excitation situation as seen in Figure 12. In case of deviation analysis in Table 3 or Figure 10, it seems the deviation of resonance frequency at the specimen #1 was not the smallest value but the relative error analysis may be a more reasonable consequence because deviations from the mean value alone cannot account for the different original resonance frequency according to the direction of carbon fiber. From the previous study [15], the zero degree of carbon fiber was selected as the best solution with respect to the structural rigidity but very sensitive for the variation of direction of carbon fiber. The test result from this study indicated that the specimen #1 has another advantage over the temperature condition by showing the smallest sensitivity result in both the resonance frequency and the modal damping coefficient. In particular, the variation of modal damping coefficient over the temperature condition is considerably robust at the specimen #1 with respect to the standard deviation.

## 4. Conclusions

The relationship between the direction of the carbon fiber and the frequency response function was experimentally identified using a simple specimen under temperature variable conditions. A result from the previous study that the sensitivity between the carbon fiber’s direction and the resonance frequency has a large effect was confirmed. To obtain the modal damping coefficient, the representative frequency response function value from the frequency response function values measured at different locations was used. Both the resonance point and the modal damping coefficient changed with temperature with great sensitivity, but the modal damping in particular showed non-linear characteristics that did not show a consistent trend depending on the temperature condition. However, it was found that specimen #1 had the highest structural rigidity under the given load condition and had relatively less change in its damping coefficient with temperature change and thus was the most robust to the temperature variable. In any case of direction of carbon fiber, the estimation of the modal damping coefficient of CFRP material should be conducted under consideration of temperature condition.

## Figures and Tables

**Table 1:** Profile of harmonic excitation.

No.	Frequency (Hz)	Acceleration (g)
1	10	0.5
2	500	0.5

**Table 2:** Temperature condition in environmental chamber.

No.	Temperature (°C)
1	−8
2	20
3	50
4	80
5	105

**Table 3:** Variation of the first resonance frequency according to different temperature conditions (Unit: Hz).

Directivity	−8 °C	+20 °C	+50 °C	+80 °C	+105 °C	S.D.
0 degrees	235.5	246.5	246.0	249.5	253.0	6.6
30 degrees	141.5	123.0	121.5	120.0	114.5	10.2
45 degrees	99.0	96.0	94.0	91.5	91.5	3.2
60 degrees	85.0	68.5	71.5	71.0	67.5	7.1
90 degrees	76.5	73.5	66.0	64.0	64.5	5.7

**Table 4:** Variation of the modal damping coefficient according to different temperature conditions (Unit: %).

Directivity	−8 °C	+20 °C	+50 °C	+80 °C	+105 °C	S.D.
0 degrees	1.6	1.2	0.9	1.0	0.9	0.3
30 degrees	18.5	2.2	1.6	0.8	5.1	7.4
45 degrees	2.4	6.1	2.4	2.1	2.4	1.7
60 degrees	2.6	13.0	3.8	2.9	8.5	4.5
90 degrees	2.7	2.0	2.4	3.1	5.7	1.5

**Table 5:** Maximum relative error over the result in a condition of −15 °C.

Specimen	Resonance Frequency	Modal Damping Coefficient
#1	6.8%	18.2%
#2	17.7%	91.3%
#3	10.0%	153.8%
#4	21.2%	559.1%
#5	18.2%	139.3%
